# Current State of Pediatric Heart Failure

**DOI:** 10.3390/children5070088

**Published:** 2018-06-28

**Authors:** Bibhuti B. Das

**Affiliations:** Joe DiMaggio Children’s Heart Institute, Memorial Health Care System, Hollywood, FL 33021, USA; bdas@mhs.net

**Keywords:** pediatric heart failure, advanced heart failure, ventricular assist device, mechanical circulatory support

## Abstract

Pediatric heart failure (HF) represents an important cause of morbidity and mortality in childhood. There is an overlapping relationship of HF, congenital heart disease, and cardiomyopathy. The goal of treatment of HF in children is to maintain stability, prevent progression, and provide a reasonable milieu to allow somatic growth and optimal development. Current management and therapy for HF in children are extrapolated from treatment approaches in adults. There are significant barriers in applying adult data to children because of developmental factors, age variation from birth to adolescence, and differences in the genetic expression profile and β-adrenergic signaling. At the same time, there are significant challenges in performing well-designed drug trials in children with HF because of heterogeneity of diagnoses identifying a clinically relevant outcome with a high event rate, and a difficulty in achieving sufficient enrollment. A judicious balance between extrapolation from adult HF guidelines and the development of child-specific data on treatment represent a wise approach to optimize pediatric HF management. This approach is helpful as reflected by the increasing role of ventricular assist devices in the management of advanced HF in children. This review discusses the causes, epidemiology, pathophysiology, clinical manifestations, conventional medical treatment, clinical trials, and the role of device therapy in pediatric HF.

## 1. Introduction

Heart failure (HF) is an important healthcare issue in both adults and children because of its high mortality, morbidity, and cost of care. By 2030, more than 8 million people in the Unites States (US) (1 in every 33) will have HF, and the projected cost estimates of treating patients will be $160 billion in direct costs as forecasted by American Heart Association (AHA) [1]. Although there is no up-to-date data for pediatric HF cost of care, it is has been reported from the Healthcare Cost and Utilization Project Kids’ inpatient database that, from 2000 to 2009, there has been an almost two-fold increase in pediatric HF related hospitalization charges [2]. The global burden of HF is unknown as too many children have no access to medical care and die of HF each year. Therefore, there is a need to develop cost-effective therapies for pediatric HF. This review summarizes and provides an outlook for evolving therapies and focus on practical issues required for management of pediatric HF.

### Definition

As per the American College of Cardiology (ACC)/AHA task force on practice guidelines published in 2005, the term “heart failure” is preferred over the older term “congestive heart failure” [3]. Heart failure in children is a clinical and pathophysiological syndrome that results from ventricular dysfunction, volume or pressure overload, either alone or in combination [4]. As a complex clinical syndrome, HF is characterized by typical symptoms and signs associated with specific circulatory, neurohormonal, and molecular abnormalities. The term “acute HF” generally describes a structural or functional alteration in the heart that occurs in minutes to hours followed by congestion, malperfusion, tachycardia, and hypotension [5]. Acute HF is not synonymous with “worsening HF”, as usually the patient has worsened either mechanically (as in the case of acute aortic or mitral insufficiency) or functionally (as a result of arrhythmia or myocardial ischemia) in pre-existing heart disease [6]. Often in patients with a diagnosis of HF, the disease progresses due to sub-optimal treatment or a lack of adherence to medical therapy, and presents with clinical decompensation, and the term “acute on chronic HF” may be used. “Chronic HF” in children is a progressive clinical and pathophysiological syndrome caused by cardiovascular and non-cardiovascular abnormalities that result in characteristic signs and symptoms, including edema, respiratory distress, growth failure, exercise intolerance, and is accompanied by circulatory, neurohormonal, and molecular derangement [7]. “Advanced HF” patients are those with clinically significant circulatory compromise who require special care, including consideration for continuous inotropic therapy, mechanical circulatory support, or heart transplantation, [8]. “End-stage HF” is the final common pathway of all forms of heart disease and may lead to therapies such as orthotopic heart, lung, or heart-lung transplantation.

It is important to distinguish “right HF” and “left HF” as the clinical management is different. There are also other nomenclatures, and HF may be described as “compensated HF” or “decompensated HF” depending upon whether end-organ perfusion is maintained. Heart failure can also be described as “systolic HF” with reduced ejection fraction, HF with preserved systolic function, which is synonymous with “diastolic HF”, and combined systolic and diastolic HF. The term “high output HF” is often used to describe cardiac or extra-cardiovascular conditions leading to volume overload and congestion. In general, a commonly accepted definition of HF has been challenging.

The International Society for Heart and Lung Transplantation (ISHLT) stratified pediatric HF into four stages (Stages A–D) as in Table 1 [4], which is useful to identify those at risk for HF and who are currently asymptomatic (Stage A) versus those on the other end of the spectrum (Stage D), who have advanced HF and, thus, would require therapeutic interventions for maintenance of end-organ function.

## 2. Incidence and Prevalence

The true global incidence and prevalence of HF in children is difficult to estimate due to lack of standard definition used for HF. The phenotype of HF also differs in congenital heart disease (CHD) and cardiomyopathies. The reported incidence of HF in children is 0.97 to 7.4 per 100,000 [9]. Heart failure-related hospitalization occurs in 11,000–14,000 children annually in the US. The greatest percentage of children with HF comes from those born with CHD, and depending upon age, 25–75% of pediatric HF patients have underlying CHD [10]. The other main cause of pediatric HF is cardiomyopathy (predominantly dilated cardiomyopathy), with an estimated annual incidence of 1 per 100,000 children in the US, Australia, United Kingdom (UK), and Ireland [11,12,13]. Recently, Shaddy et al. have described the incidence of HF ranging from 0.87/100,000 (UK and Ireland), 7.4/100,000 (Taiwan), and 83.3/100,000 (Spain) [14]. Key findings of their study included a prevalence of HF associated with cardiomyopathies ranging from 36.1% (Japan) to 79% (US); associated with CHD from 8% (Norway) to 82.2% (Nigeria); associated with rheumatic heart diseases from 1.5% (Turkey) to 74% (Zimbabwe); associated with renal disorders from 3.8% (India) to 24.1% (Nigeria); and associated with HIV from 1% (US) to 29.3% (Brazil).

## 3. Etiology

**Figure 1:**
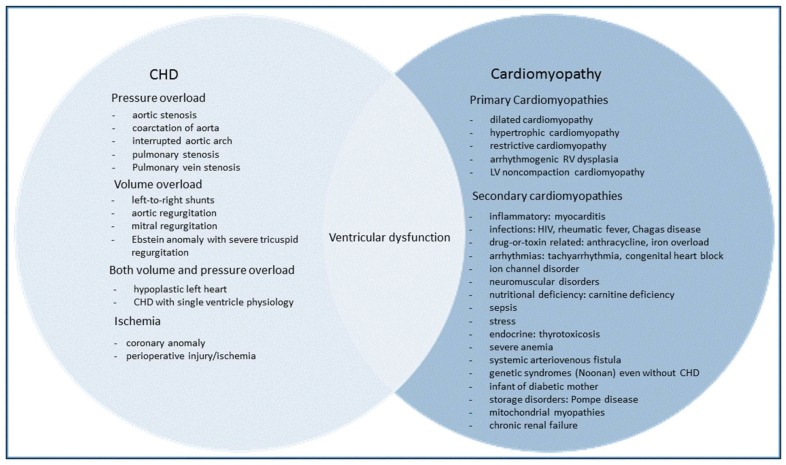
Common causes of heart failure in children—the relationship of ventricular dysfunction to CHD and cardiomyopathy.

The etiology of HF in children plays a key role in the clinical course and outcome. The two most common causes of pediatric HF are CHD and cardiomyopathies. There is an inherent problem in classifying HF based on clinical presentation of CHD when described primarily in physiological terms. For example, outflow tract obstruction (pressure overload) or pulmonary over-circulation (volume overload) may be confusing because ventricular dysfunction may be associated with poor contractility (systolic dysfunction) or poor relaxation (diastolic dysfunction), with or without the clinical presence of HF. There is an overlapping relationship of HF, CHD, and cardiomyopathy [15]. Of note, patients with CHD and HF, especially with single ventricle physiology, make up a significant proportion of the heart transplantation population, over 50% in infants [10]. The common causes of HF in children are shown in Figure 1.

## 4. Pathophysiology

In HF, low cardiac output leads to reduced baroreceptor stimulation, resulting in the activation of the sympathetic nervous system (SNS) which increases heart rate, cardiac contractility, and vasoconstriction. These mechanisms provide helpful support for the heart as a transitory compensation when the myocardium starts to fail [16]. Prolonged activation of SNS increases afterload due to sustained vasoconstriction and renin release. The release of renin leads to the production of angiotensin II, which, in turn, causes vasoconstriction of renal afferent arterioles, as well as the release of aldosterone, which causes an increase in sodium reabsorption. When the renin-angiotensin-aldosterone system (RAAS) activation is undeterred, vasoconstriction (increased afterload) leads to cardiomyocyte hypertrophy and apoptosis [17]. In addition, RAAS has an important profibrotic effect on cardiac tissue and promoting endothelial dysfunction. 

**Figure 2:**
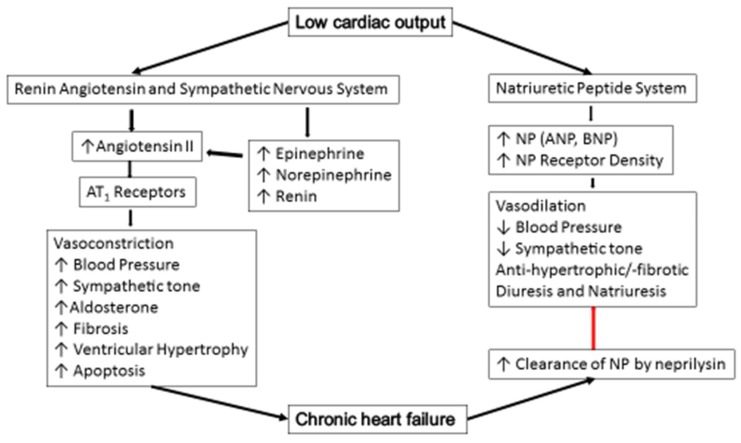
Pathophysiology of chronic heart failure. (AT_1_ = angiotensinogen 1, ↑ = increase, ↓ = decrease, NP = natriuretic peptide, ANP = atrial natriuretic peptide, BNP = B-type natriuretic peptide).

At the cellular level, the compensatory gain in cardiac excitation-contraction coupling mediated by sympathetic stimulation ultimately becomes unsuccessful, as the sustained leak of calcium from the sarcoplasmic reticulum leads to depletion of intracellular calcium and ultimately impairs contractility [18]. Unfortunately, the deleterious effects predominate over the long-term, leading to pathologic myocardial remodeling, and more rapid progression of myocardial dysfunction (Figure 2).

On the other hand, there are several peptides, such as the natriuretic peptides (NPs), bradykinin and adrenomedulin, that help to ameliorate all the harmful effects of SNS and RAAS by attenuating vasoconstriction, sodium retention, and retarding cardiac and vascular remodeling. Natriuretic peptides are coupled to, and activate, guanyl cyclase A, which increases the intracellular concentrations of the second messenger, cyclic guanosine monophosphate. The latter, in turn, activates protein kinase G, leading to vasorelaxation, natriuresis, and diuresis. Atrial natriuretic peptide and B-type natriuretic peptide (BNP) also inhibit renin secretion and aldosterone production and attenuate cardiac and vascular remodeling, apoptosis, ventricular hypertrophy, and fibrosis [19]. Normally, these compensatory actions are not sufficient to prevent or stop HF development because NPs are readily destroyed by an enzyme, neprilysin. Neprilysin levels are increased in chronic HF, and, thus, the clearance of these neuropeptides is accelerated [20]. In addition, RAAS is responsible in mediating renal hypo responsiveness to NPs, which facilitates progression of HF [21].

## 5. Clinical Presentation

The clinical picture of HF is directly related to age [22]. Table 2 describes the age-related specific signs and symptoms due to HF. In general, the symptoms of HF depend upon whether there is congestion due to chronic right HF or hypo-perfusion due to acute left HF. Signs and symptoms of chronic right HF include elevated jugular venous pressure, pleural effusion, ascites, pedal edema, abdominal discomfort, and hepatomegaly. Signs and symptoms of acute left HF include dyspnea, orthopnea, rales on auscultation due to pulmonary edema, dizziness, fatigability, nausea, vomiting, abdominal pain, and feeding intolerance. When right HF is acute it can present with hypo-perfusion, tachycardia and hypotension. Similarly, when left HF is chronic it can present with signs and symptoms of chronic congestion. Right HF associated with left HF and is a predictor of increased morbidity and mortality.

The well-known New York Heart Association (NYHA) HF classification does not apply to young children on a practical level and is thought to lack the sensitivity needed to assess and capture the progression of HF severity in children. For this reason, the modified Ross HF classification [23] is used for the assessment of children younger than six years with HF. A comparison of two classifications is shown in Table 3.

**Figure 3:**
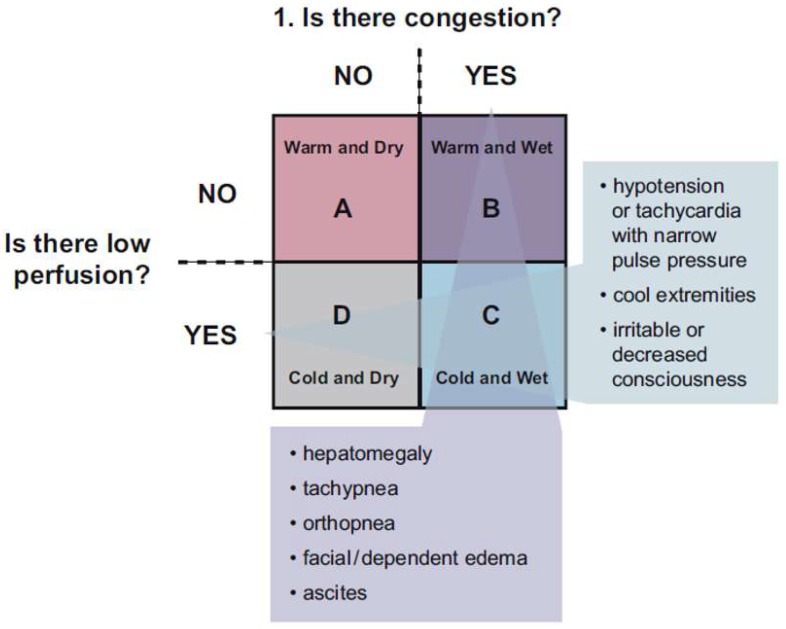
Perfusion and congestion model: patterns of presentation in heart failure in children [22]. (Presented with permission from the Publisher, Elsevier, originally published in Canadian Journal of Cardiology 2013; 29:1535-1552) (License Number 4335401450214).

If a patient presents with symptoms of HF, further classification into perfusion (warm vs. cold) and congestion (filling pressure) status (dry vs. wet) provides a current clinical context with the ultimate goal being warm and dry (well perfused with normal filling pressure). Figure 3 illustrates a useful construct to assist the clinician in their evaluation, based on the presence of abnormal perfusion and increased fluid congestion [22]. Recently, an analysis from the Pediatric Heart Transplant Society database described that (I) congestion is more common than low cardiac output/hypoperfusion in children with end-stage HF and correlates with NYHA/Ross classification and end-organ dysfunction; (II) the severity of HF symptoms based on NYHA/Ross classification correlates best with elevations in pulmonary capillary wedge pressure (defined as >15 mmHg); (III) end-organ function correlates best with elevation in right atrial pressure; and (IV) death or deterioration while waiting for heart transplant is highest among children with both congestion and low cardiac output [24]. Another study by Mullens et al. has also suggested a primary role of congestion in the pathophysiology of end-organ dysfunction occurring in the setting of chronic left HF [25]. Children with both congestion and hypoperfusion (wet/cold) have the highest risk of death or clinical deterioration.

## 6. Diagnostic Approach

**Figure 4:**
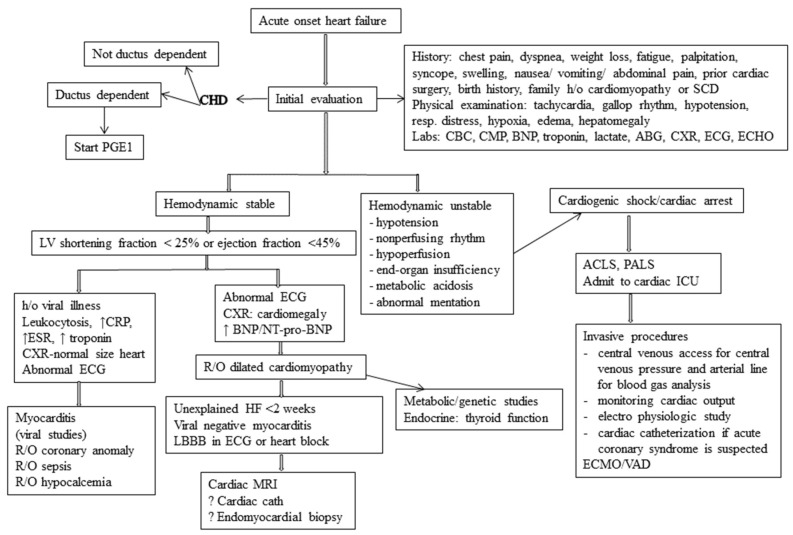
Diagnosis and management of acute heart failure in children. (CHD = congenital heart disease, PGE = prostaglandin E, SCD = sudden cardiac death, CBC = complete blood count, CMP = comprehensive metabolic panel, ABG = arterial blood gas, CXR = chest-X-ray, ECG = electrocardiogram, ECHO= echocardiogram, ACLS = advanced cardiac life support, PALS = pediatric advanced life support, ICU = intensive care unit, ECMO = extra-corporeal membrane oxygenation, VAD = ventricular assist device, MRI = magnetic resonance imaging, CRP = C-reactive protein, ESR = erythrocyte sedimentation rate, R/O = rule out, LBBB = left bundle branch block, BNP = B-type natriuretic peptide).

The first step in the diagnostic approach to pediatric HF is history and physical examination. Laboratory investigations should include: complete blood count to assess anemia and rule out infections, arterial blood gas, and electrolytes to evaluate hyponatremia, hyperkalemia, hypoxemia and acidosis, renal/hepatic function and lactate to evaluate end-organ function, natriuretic peptides (NT-pro-BNP/BNP) to evaluate cardiac function and LV filling pressure, troponin to rule out any inflammatory or ischemic cardiomyopathy, chest radiography to evaluate cardiac size and pulmonary edema, electrocardiogram to rule out arrhythmia/ischemia/left bundle branch block, echocardiography to determine any structural or functional alteration of heart, cardiac magnetic resonance imaging to evaluate for specific forms of cardiomyopathy and to evaluate complex CHD, and cardiac catheterization to evaluate hemodynamic parameters and cardiac output, especially in complex CHD. The echocardiogram is particularly useful because it is widely available and provides the bedside information on cardiac systolic/diastolic function, chamber volumes/diameters, wall thickness, right ventricular function, and pulmonary pressure. These data are crucial to make correct diagnosis and to guide appropriate management. A proposed practical approach to a pediatric patient presenting with acute systolic HF is shown in Figure 4.

## 7. Medical Therapy

The goals of acute HF management in children are to improve hemodynamics and prevent progression. Current management includes stabilization with intravenous inotropes/vasopressors, mechanical ventilation, treatment of arrhythmia, and progression to mechanical support, if needed. Should some recovery occur, the child is often left with chronic HF and the lingering diagnosis of dilated cardiomyopathy, with or without a genetically-based or syndrome/systemic disease-based diagnosis. The goal of clinical management of chronic HF in children is to maintain stability, prevent progression, and provide a reasonable milieu to allow somatic growth and optimal development. Despite the lack of sufficient randomized prospective studies, angiotensin-converting enzyme inhibitors (ACEi) are first-line, and β-receptor antagonists are second-line therapies in children. Following the adult guidelines, and without having data pertaining to the pediatric population, mineralocorticoids are also accepted in the treatment of pediatric HF, while diuretics should only be used to achieve a euvolemic status. The common drugs used in pediatric HF with their doses and mechanism of actions are summarized in Table 4.

Current treatment for chronic HF includes some combination of ACEi (level of evidence B), β-blockers (level of evidence B), diuretics (level of evidence C), aldosterone antagonists (level of evidence C), and digoxin (level of evidence C) [4]. Diuretics are used to treat fluid retention associated with ventricular dysfunction, ACEi decrease afterload by antagonizing the renin-angiotensin aldosterone system, β-blockers antagonize the deleterious effects of chronic sympathetic myocardial activation and can reverse LV remodeling and, additionally, carvedilol has vasodilatory properties due to its additional α-blocking action, while ACEi and β-blockers can slow disease progression and prolong survival, titration and tolerability often present challenges. The routine use of anticoagulants in patients with systolic heart failure and no history of thromboembolic events is not recommended (level of evidence C) [4]. Inotropic therapy may be considered for symptomatic relief in the palliative setting, but the use of inotropes for chronic therapy in children with HF other than as bridge to transplant is not recommended (level of evidence C) [4].

When deciding on an outpatient medical therapy for pediatric HF, the two most often relied upon factors include efficacy shown in adult HF trials, and pediatric consensus statements and guidelines. Although, the management of pediatric HF has been extrapolated from adult trials, there are significant barriers in applying adult data to children because of developmental factors, age variation from birth to adolescence, and differences in the genetic expression profile and β-adrenergic signaling [26,27]. 

## 8. Clinical Trials and Their Outcomes

There is a paucity of randomized prospective clinical trials in pediatric HF from which a strong evidence-based recommendation (level of evidence A) regarding pharmacotherapy can be made. Although there are many reasons for this, the significant challenges inherent in performing well-designed drug trials in children, especially in cardiac research, are heterogeneity of diagnoses, identifying a clinically relevant outcome with a high event rate, and insufficient enrollment [28]. An example of the difficulty of performing a prospective study in the pediatric population is given from the “Pediatric Heart Network ACE inhibition in mitral regurgitation study”, which was terminated 17 months after initiation. In this study, 349 children were screened, and only five were enrolled [29]. Currently, few randomized, controlled trials in pediatric HF exist and are summarized in Table 5.

In 1993, a study to assess the effect of treatment with ACEi over digoxin and diuretics on survival of pediatric patients with chronic HF due to dilated cardiomyopathy demonstrated that patients treated with ACEi have a beneficial survival effect, similar to observations in adult patients [30]. The first and only randomized, controlled study in pediatric HF, which addressed the use of carvedilol for HF management [31]. This study, however, was underpowered and included the full range of pediatric patients (infancy through 18 years of age) with CHD (including single ventricle patients with failing systemic right ventricle), as well as patients with normal cardiac anatomy and systolic LV failure, and failed to show any benefit of the treatment, unlike the adult studies. In spite of the negative outcomes, that study highlighted a number of issues faced in pediatric cardiac research, including the underpowered study, the difficulties in interpretation of results from a heterogeneous population, and the importance of design of the composite end-point. Several retrospective studies showed that carvedilol improved the ejection fraction and improved the HF clinical status in children [32,33].

A retrospective study in 2010 detected a positive benefit of ACEi in children with dilated cardiomyopathy compared to no treatment; however, treatment with digoxin/diuretic revealed comparable results [34]. The largest prospective study in children with single ventricle physiology with right ventricle as systemic ventricle and preserved ejection fraction, by Hsu et al. [35]. This study has not proven a clinical benefit of ACEi treatment; however, these data cannot be transferred to children with cardiomyopathy and reduced systolic function. Despite the lack of prospective, randomized, blinded, placebo-controlled trials in children with dilated cardiomyopathy, treating children with HF with ACEi is widely accepted and is also recommended by ISHLT for the management of HF (level of evidence B) [4].

There are several promising medications which will be available in the near future and which give hope for pediatric HF patients, especially for the management of chronic HF. Ivabradine, an I_f_ current inhibitor in the sinoatrial node acts by reducing heart rate and is found to be helpful in reducing HF hospitalization and death from HF in children [36]. The PARADIGM-HF (Prospective comparison of angiotensin receptor antagonist (Valsartan) and neprilysin inhibitor (Sacubitril) with angiotensin converting enzyme inhibitor (Enalapril) to determine impact on Global Mortality and Morbidity in Heart Failure) trial, has demonstrated that Sacubitril/Valsartan is superior to Enalapril in reducing the risks of both sudden cardiac death and death from worsening heart failure [37]. This novel combination of drugs, Sacubitril/Valsartan is also shown to reduce the risk of hospitalization and the progression of heart failure in adults. However, the benefit of Sacubitril/Valsartan in pediatric heart failure patients is unknown.

The current pediatric multicenter trial (PANORAMA-HF study, NCT00382525) will examine whether the combination of Sacubitril and Valsartan is superior to Enalapril for the treatment of HF in pediatric patients with reduced systemic LV systolic function. The details of the design of the trial has been recently published, and notably the study population is quite homogenous, including only patients with reduced LV systolic function [38]. The target N is 180 patients in each treatment group (Enalapril vs. Sacubitril/Valsartan), and though far from the vast populations studied in adult HF trials, is expected to provide adequate power to detect a meaningful difference in an event-driven endpoint (primary global rank endpoint). The end-point in this trial may be the key element in the success of pediatric HF research in the future, as the use of mortality as an endpoint is unlikely to be an achievable goal given the number of patients required.

## 9. Resynchronization Therapy

Cardiac resynchronization therapy (CRT) is an effective treatment for adult patients [39]. However, information on the outcome of pediatric CRT is limited. Interestingly, dilated cardiomyopathy was shown to be an independent risk factor for non-response to CRT in children, which is in contrast to the adult population [40]. In most children with dilated cardiomyopathy, the QRS duration does not meet the adult criteria for CRT. Several case reports, as well as a limited number of multicenter retrospective trials, have reported an improvement in functional status with biventricular pacing or multisite pacing in children with HF, but no long-term trials have been performed to establish the efficacy of CRT [41]. CRT is not favorable for all children, however, it should be considered if a child with severely-reduced LV systolic dysfunction (LV EF < 35%) with prolonged QRS duration, and with or without complete left bundle branch block [42]. Permanent pacemaker implantation is recommended for advanced second- or third-degree atrioventricular block with ventricular dysfunction (level of evidence B) [4]. Recently, remote monitoring of pacemaker function in children has been shown to be successful [43].

## 10. Mechanical Circulatory Support and Heart Transplantation

Medical therapy has improved the survival and quality of life of children with HF; however, there is still a significant proportion of patients who have poor outcomes and need advanced HF therapy, including continuous intravenous inotropes, mechanical support, and/or heart transplantation. Heart transplant offers prolonged survival for children with end-stage HF; however, cardiac transplantation is a last resort, given the limited availability of donor organs, complicated management and associated morbidity and mortality [44]. The wait list mortality is less than the transplantation rate (17% vs. 63%), and the number of children receiving heart transplants for all diagnoses is about 600 patients annually worldwide [45]. 

**Figure 5:**
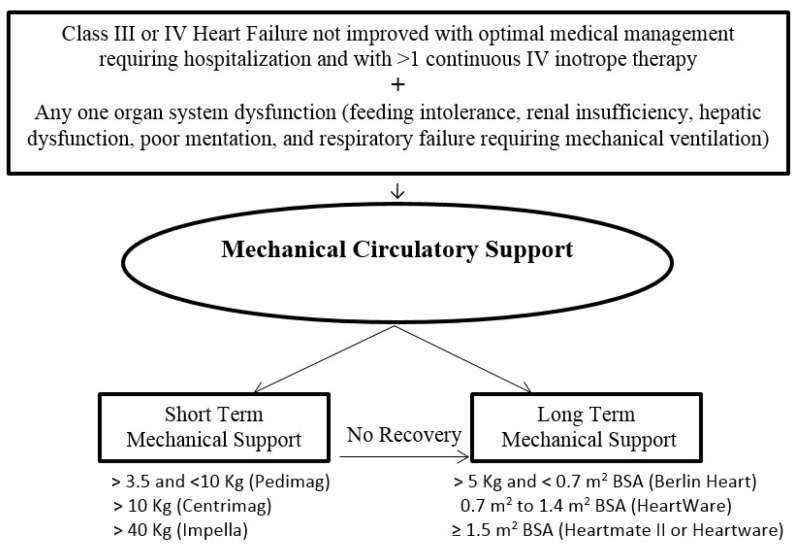
Indication of mechanical circulatory support and device selection in children with heart failure. (BSA = body surface area, IV = intravenous).

Mechanical circulatory support systems should be used in children with decompensated HF who cannot be stabilized with medical therapy alone to unload the failing ventricle and maintain end-organ perfusion. The AHA guidelines for recommendations for use of mechanical circulatory support, device strategies, and patient selection are available for adults [46]. However, there are no published guidelines for determining the appropriateness of mechanical circulatory support in children with HF. A simplified general approach for indication for mechanical circulatory support and the selection of a device in children is described in Figure 5.

Until recently, extracorporeal membrane oxygenation (ECMO), which provides total cardiopulmonary support, was the only modality available to support children with end-stage HF. However, ECMO has been shown to be associated with high waitlist mortality and poor post-transplant survival [47]. This is particularly true for infants <1 year of age, for those with a diagnosis of complex CHD and for patients with renal insufficiency. The landmark prospective Berlin Heart EXCOR (Berlin Heart Inc., Berlin, Germany) Trial [48], which compared patients supported on the Berlin Heart ventricular assist device (VAD) with a historic matched cohort of patients supported on ECMO, led to US Food and Drug Administration approval of the device in 2011. Since then, use of VAD as a mechanical circulatory support in children has increased from 4% in the Blume et al. [49] study in 2006 to 22% in the more recent years, with a successful bridge to heart transplantation in 96% of children and post-HT survival comparable to children not needing VAD support [50].

Pediatric VADs offer specific advantages over ECMO, including reduced anticoagulation requirements, decreased use of blood products, decreased risk of systemic thromboembolic complications and inflammatory response, longer duration of support, increased mobility, participation in rehabilitation, and the possibility of hospital discharge [51,52]. Although VAD support and durability are superior compared to ECMO, a high rate of serious adverse events persist, including infection, bleeding, device malfunction, and neurologic injury in children [53,54]. Some VADs (Pedimag (Thoratec Corp., Pleasanton, CA, USA), Centrimag (Thoratec Corp., Pleasanton, CA, USA), and percutaneous VAD (Impella) (Abiomed, Danvers, MA, USA)) are specially designed to provide short-term or temporary circulatory support in acute hemodynamic compromise while more definite treatment decisions are made. The use of short-term VADs in children with decompensated HF has demonstrated longer survival to transplant compared to ECMO [55]. The Berlin Heart is a paracorporeal pulsatile VAD that can be used for left, right, or bi-ventricular support in a child as small as 3 kg. Intracorporeal continuous flow devices have replaced pulsatile paracorporeal VAD in larger children and adolescents. Long-term support with durable VADs, such as HeartMate II (Thoratec Corp., Pleasanton, CA, USA), and HeartWare HVAD (Heartware Inc., Framingham, MA, USA), are available, but it is important to note that these devices are designed for adults with structurally normal hearts. Smaller implantable devices have been available since US FDA approval of EXCOR PVAD in 2011, such as the HeartWare HVAD, which can be used as a bridge to transplant in children down to a body surface area of 0.7 m^2^. 

Although the availability of smaller pulsatile and continuous flow VAD devices have allowed young children to be successfully supported, children with CHD, especially with single ventricle physiology, posing unique challenges in configuring ideal cannulation and maintaining adequate systemic oxygenation and cardiac output. It is now recognized that an increasing number of single ventricle CHD subjects who have survived initial palliative surgery will experience HF and end-organ dysfunction due to intrinsic inadequacies of a circulation supported by a univentricular heart. The survival to heart transplant of children with functional single ventricles supported with VADs is 47.5% and one-year post-transplant survival outcomes are similar with bi-ventricular CHD patients who are on a VAD at the time of transplant [56]. Successful use of the Total Artificial Heart (SynCardia Systems, Inc., Tuscon, AZ, USA) in children with HF due to complex CHD has also been reported [57].

## 11. Conclusions

Pediatric HF is a complex clinical syndrome resulting from diverse primary and secondary causes and shared pathways of disease progression, correlating with substantial mortality, morbidity, and cost. Treatment of pediatric HF has evolved over the past decade to meet the growing demands and challenges in the care of this complex group of patients. The ultimate goal is to find a readily available, affordable, easily administered and safe therapy that will stall the progression of HF indefinitely, concomitantly allowing reasonably normal growth and development, however, this has been an illusion to date. Early identification and selective treatment of patients who are stage A/B is critical to prevent progression to end-stage HF (stage D). Although clinicians have come a long way in the implementation of life-prolonging therapies in pediatric HF, with the rising cost of therapy, preventative strategies and efficiency of care are needed. The emerging evidence from the recent studies in pediatric HF and a collaborative multidisciplinary approach will likely to improve the outcomes of children with HF in future.

## Figures and Tables

**Table 1:** Heart failure staging in children.

Stage (Class)	Description
A	Patients with increased risk of developing HF but with normal cardiac function and chamber size. Examples: univentricular heart, previous exposure to anthracycline, Duchenne muscular dystrophy, congenitally corrected transposition of the great arteries, h/o familial dilated cardiomyopathy
B	Patients with abnormal cardiac morphology or function with no past or current symptoms of HF. Examples: asymptomatic dilated LV, isolated LV noncompaction
C	Patients with past or current HF symptoms and structural or functional heart disease
D	Patients with end-stage HF requiring continuous infusion of inotropic agents, mechanical circulatory support, cardiac transplantation, or hospice care

HF = heart failure, LV = left ventricle.

**Table 2:** Common signs and symptoms of heart failure in children.

Infants	Toddlers
Growth failure	Respiratory distress
Persistent tachypnea	Poor appetite
Hepatomegaly	Decreased activity
Respiratory distress	Hepatomegaly
**School Age**	**Adolescents**
Fatigue	Chest pain
Exercise intolerance	Dyspnea
Poor appetite	Pain abdomen, nausea/vomiting
Hepatomegaly	Hepatomegaly
Orthopnea	Orthopnea

**Table 3:** NYHA and modified Ross classification of heart failure in children.

Modified Ross Classification of HF in Children < 6 year	NYHA Classification of HF in Children > 6 year
Class I: Asymptomatic	Class I: Asymptomatic
Class II: Mild tachypnea or diaphoresis with feeding in infants; dyspnea on exertion in older children	Class II: Slight or moderate limitations of physical activity
Class III: Marked tachypnea or diaphoresis with feeding in infants. Prolonged feeding times with growth failure; Marked dyspnea on exertion in older children	Class III: Marked limitation of physical activity
Class IV: Symptoms such as tachypnea, retractions, grunting, or diaphoresis at rest	Class IV: Symptoms at rest.

**Table 4:** Conventional pharmacotherapy of chronic heart failure in children.

	Standard Dose	Mechanism of Action	Comments
**Diuretics**			
1. Furosemide	1 mg/kg dose BID up to max 6 mg/kg/day	Relieves congestive symptoms; useful in volume overload/fluid retention states; do not change the long-term outcomes	Aggressive use of diuretics can reduce the preload and may result in neurohormonal activation and fluid retention—a vicious cycle; patients refractory to usual oral dose of diuretics may need IV diuretics to relieve congestion
2. Chlorothiazide	10 mg/kg dose BID up to max 2 gm/day		
3. Metolazone	0.1 mg/kg dose BID up to max 20 mg/day		
Digoxin	3 to 5 mcg/kg dose BID	Increases inotropy; attenuates neurohormonal activation that results in decreased serum norepinephrine, improves baroreceptor function, decreases sympathetic nervous system activity	Very narrow toxic to therapeutic ratio; most common side effects are conduction disturbances (atrioventricular block); useful for atrial arrhythmia; reduces inter-stage mortality in infants with single ventricle CHD; excreted by the kidney so the dose must be decreased with renal insufficiency
**ACE inhibitors**			
1. Captopril	0.1 mg/kg dose TID up to max 2 mg/kg/dose	Decreases mortality and morbidity; blocks the conversion of angiotensin I to II and activates bradykinin and kallidin; causes vasodilation and natriuresis; reduces afterload	ACE inhibitors are beneficial in ISHLT HF stage B to D HF patients; not recommended for asymptomatic children with mild ventricular dysfunction, no recommendation for routine use in single ventricle CHD patients with RV as systemic ventricle; side effects include hypotension, and renal insufficiency
2. Enalapril	0.1 mg/kg dose BID up to max 0.5 mg/kg/day		
**Beta-blockers**			
1. Metoprolol	0.1 mg/kg dose BID up to max 1 mg/kg dose	Decreases morbidity and mortality; Carvedilol has vasodilatory, antioxidant, antiproliferative and anti-apoptotic properties, reversing cardiac remodeling	Patients with ISHLT HF stage C and D; may be beneficial in children with HF due to CHD when LV is systemic ventricle; because of downregulation of β-2 receptor in children with HF due to dilated cardiomyopathy—a better option may be Metoprolol
2. Carvedilol	0.025 mg/kg/dose BID up to max 0.5 mg/kg/dose BID		
Aldosterone antagonist Spironolactone	1 mg/kg dose BID up to max 200 mg/day	Decreases mortality and morbidity; improves endothelial dysfunction; suppresses vascular angiotensin conversion	Should be used with caution in patients with hyponatremia, renal insufficiency, hyperkalemia and hepatic disease; can cause gynecomastia

BID = twice daily, TID = three times daily, max = maximum, LVEDP = left ventricular end-diastolic pressure.

**Table 5:** Summary of important pediatric heart failure clinical trials.

Title	Journal/Year (Reference)	Key Findings
Effect of treatment with ACE inhibitor on survival of pediatric patients with dilated cardiomyopathy	Ped Cardiol, 1993 [30]	*n* = 80; (26 = ACEi, 54 = digoxin plus diuretics). One-year and two-year survival with ACEi was better than digoxin group
Carvedilol for children and adolescent with heart failure. A randomized control trial	J Am Med Assoc, 2007 [31]	*n* = 161; No significant difference between treatment vs. placebo group in primary end point (clinical improvement) or secondary end point (ventricular function or serum BNP)
Safety of enalapril in infants with SV CHD with RV as systemic ventricle, multicenter randomized trial	Circulation, 2010 [35]	*n* = 230; No improvement in somatic growth, ventricular function, heart failure severity. Routine use of Enalapril in SV CHD patients with RV is not recommended as systemic ventricle
Ivabradine in children with dilated cardiomyopathy and symptomatic chronic HF trial: randomized, double-blind, placebo-controlled trial with 12-months follow-up	J Am Coll Cardiol, 2017 [36]	*n* = 116; Primary end-point reached by 51 of 73 children taking Ivabradine (70%); Ivabradine safely reduced the resting heart rate of children with chronic HF and dilated cardiomyopathy; improvement in ejection fraction, functional class, and NT-pro-BNP was noted.

BNP = B-type natriuretic peptide, ACEi = angiotensin converting enzyme inhibitor, CHD = congenital heart disease, SV = single ventricle, *n* = number; HF = heart failure.

## Abbreviations

HF	Heart failure
CHD	Congenital heart disease
SV	Single ventricle
LV	Left ventricle
RV	Right ventricle
VAD	Ventricular assist device
NYHA	New York Heart Association
BNP	B-type natriuretic peptide
ACE	Angiotensin converting enzyme
